# Human-inspired sensorimotor controller for dynamic motion adaptation: a study in robotic arms

**DOI:** 10.3389/fnbot.2026.1821320

**Published:** 2026-05-22

**Authors:** Maxime Marchal, Raphaël Furnémont, Tom Verstraten, Ghilés Mostafaoui

**Affiliations:** 1 Brubotics, Vrije Universiteit Brussel, Elsene, Belgium; 2 Flanders Make, Lommel, Belgium; 3 ETIS, CY Cergy Paris Université, Cergy, France

**Keywords:** biomimetic control, dynamic task adaptation, human-inspired controller, robotic arm control, sensorimotor learning

## Abstract

Robotic systems operating in dynamic environments often struggle to adapt their movements to external sensory signals without relying on explicit analytical models or conventional position-tracking control schemes. This limitation is especially problematic in systems where detailed dynamic models or positional feedback are limited or unreliable. Addressing this gap requires control architectures that, like human sensorimotor systems, can learn and generalize from experience without requiring an accurate model of the system's dynamics. In this paper, we present a human-inspired controller based on sensorimotor learning for articulated robotic arms. Departing from conventional control strategies relying on precise dynamic models, this approach emphasizes adaptability to dynamic tasks and external stimuli while accounting for gravity and system dynamics. The controller operates in two phases: an offline exploration phase that builds a discretized database of system responses for sampled initial states and actuator current commands, and an online exploitation phase that selects control actions by matching the current state to the closest explored state and applying a probabilistic command-selection rule. Rather than relying on an explicit analytical dynamics model, the approach uses empirical state–action–response relationships learned from the system's own behavior. During exploitation, the current joint positions and velocities are used to identify the closest explored state, while the command selection itself is based on velocity-response distributions learned during exploration. The controller's evaluation on various dynamic trajectories, including sinusoidal and trapezoidal profiles, demonstrates the feasibility of the framework on a 2-DOF RR robotic arm. Further analysis investigates the influence of control time parameters, the impact of actuator friction, and the controller's redeployability to modified mechanical configurations after re-exploration. These results underline the potential of biologically inspired learning mechanisms for data-driven robotic control while also highlighting limitations related to discretization, scalability, and future online adaptation.

## 1 Introduction

With the growing development of socially and physically interactive robotics, it is crucial to design control systems that enable intuitive and efficient interactions while minimizing cognitive load for humans. A key requirement is ensuring that robots can adapt to external sensory signals, such as the movement dynamics of a human partner. This challenge begins with understanding human sensorimotor control and developing models that allow robots to emulate the intuitive and adaptive nature of human motion.

Human motor control system enables performing a wide variety of motions (Latash et al., 2016). Among them, we can cite two prominent families: Rhythmic and discrete movements (Schaal, 2006). Many studies tend to identify the differences and similarities between the generations of such movements. Some authors, such as Hogan and Sternad (2007), have proposed a method to classify the different gestures. They define a discrete movement as a movement that occurs between two postures. Rhythmic movements are themselves categorized into four subgroups, ranging from strictly periodic movements to movements with recurring patterns. However, the two definitions are not exclusive. Indeed, rhythmic movement can be performed between two postures, and a discrete movement can be repeated periodically. The biological underlying mechanisms responsible for generating such rhythmic and discrete movements are still poorly understood and are the subject of much debate (Nordin et al., 2017; Degallier and Ijspeert, 2010).

In addition to being able to generate a wide range of movements, humans possess a remarkable ability to adapt their actions to new and dynamic environments (Kamboj et al., 2024). The adaptation of movement dynamics to external stimuli is at the center of this article's interest. In humans, they have been analyzed at different levels, including neurobiology, behavioral studies, and computational modeling of human motor control.

Behavioral studies on interpersonal coordination have shown that humans can dynamically synchronize their bodies with each other thanks to information exchange (Richardson et al., 2005). This information exchange can occur through visual (Schmidt et al., 2007), tactile (Lenay, 2010), or auditory information (Shockley et al., 2007). For instance, in terms of visual information, several articles have shown that some visual cues are better suited to establish interpersonal coordination. Participants exhibited greater unintentional coordination and more stable intentional coordination with a stimulus when they tracked it with their eyes (Schmidt et al., 2007). Furthermore, unintentional coordination is weaker when a moving partner is in peripheral view compared to when they are in central view (Richardson et al., 2007). Finally, Hajnal et al. (2012) found that the amount and location of available visual information influence the stability of the observed synchronization.

From a neurobiological point of view, many studies have focused on the role of the spinal cord and the Central Pattern Generator (CPG), particularly in the case of locomotion (Forssberg, 1985). Some authors have hypothesized that these control units (CPG) could be used in the case of rhythmic arm movements in humans (Zehr et al., 2004). At the cortical level, Schaal et al. (2004) investigated the differences between rhythmic and discrete movements using functional magnetic resonance imaging. Brain activity recordings showed that discrete wrist movements triggered activation of a higher number of brain areas, particularly those related to movement planning, than rhythmic movements. Therefore, the authors concluded that the neural circuits that enable the generation of rhythmic movements may differ from those that enable discrete movements. In Churchland et al. (2012), the authors recorded a significant rhythmic component in the motor cortex output during grasping movements, suggesting a possible oscillatory control of muscle commands. The role of the cerebellum and basal ganglia in controlling rhythmic movements has also been highlighted in recent studies (Kameda et al., 2023).

All these neurobiological and behavioral studies on rhythmic adaptations in humans highlight both the complexity of the underlying mechanisms and the uncertainties that remain regarding the nature of the signals involved in muscular control (whether they are discrete or oscillatory), the sensory information utilized (positions, velocities, and/or accelerations), and, more broadly, the organization of the movement planning and control circuits in humans. Nevertheless, many computational models of human motor control have been proposed and developed. Among the models that are the most widely used in the literature, we can find optimal control, reinforcement learning, and probabilistic models (Desai, 2015).

In optimal control, the goal is to find the control signals that achieve a desired result while minimizing a cost function (Lewis et al., 2012; Guigon, 2023). The main limits of optimal control are that it requires sufficient knowledge of the system and its uncertainties (Gerdts, 2012), it induces a curse of dimensionality (Ivaldi et al., 2012), and it is necessary to adapt the cost function if the task or environment is modified (Todorov and Jordan, 2002).

In another way, reinforcement learning consists of experimentally learning a system's behavioral strategy (Kuo et al., 2023). This is achieved by learning a policy within an environment where certain actions yield rewards (Bjelonic et al., 2023). Traditionally, reinforcement learning is based on a Markov Decision Process. The policies can be either deterministic or stochastic. There are two principal approaches to reinforcement learning: model-free and model-based methods (Sutton and Barto, 2020). However, as in optimal control, depending on the chosen approach, it also suffers from limitations such as the curse of dimensionality (Kober et al., 2013), the difficulty in defining a reward function (Sorg et al., 2010) and the risk of finding a local optimal policy (Zhang et al., 2022).

An alternative approach involves the use of probabilistic models, with Bayesian decision theory as a key example (Kording and Wolpert, 2006). This theory can be subjectively interpreted as a model of decision making in an uncertain environment. It can be divided into 3 phases (Wolpert, 2007). First, Bayesian probabilities are used to establish a relationship between the actions and the distribution of the reachable states. Then, a utility function is defined to quantify the value of taking each possible action of each possible state. Finally, the first two phases are combined to generate a decision based on a defined criterion. As for the first two methods, this approach also suffers from the curse of dimensionality (Chandra et al., 2023). Moreover, the selection of the utility function is not straightforward.

Therefore, one can see that the question of modeling rhythmic adaptations in humans remains open. The above methods have all their advantages and disadvantages (depending mainly on the task to be carried out), but are difficult to implement on real physical systems such as robots. Indeed, robotic dynamics models are often inaccurate. The identified robot's dynamic parameters are often inconsistent with the real physical parameters (Song and Hu, 2023). In addition, friction in robotic arms is highly nonlinear and difficult to model, leading to a mismatch with the real world (Źabiński and Turnau, 2005). Finally, elements in robotic arms, such as bearings, can have highly nonlinear behavior due to their defects, making their dynamics very difficult to model (Ruan et al., 2022). For all those reasons, using controllers that require perfect knowledge of system dynamics is difficult in this context (Liang et al., 2018). Moreover, most of the robots controlled by classical control methods can show limits in terms of adaptability and computational cost (Spong and Fujita, 2011). However, recent studies demonstrated that Human-Robot and Human-Human unintentional and intentional interpersonal coordination are similar if the robot controller emulates human behavior (Mostafaoui et al., 2022).

Consequently, we propose in this article to implement a sensorimotor controller inspired by the characteristics of rhythmic adaptations in humans on an articulated robotic arm of RR type. This human–inspired control strategy reflects a biomimetic perspective, in which principles observed in biological sensorimotor systems, such as experience–based learning and rhythmic adaptation are translated into robotic control. By relying on biologically plausible mechanisms rather than explicit dynamic models, the proposed controller aims to enhance adaptability and efficient motion regulation in dynamic environments. The present study is intentionally limited to a 2-DOF RR manipulator so that the discretized exploration remains tractable.

The main objectives of this human-inspired controller are:

1. Demonstrate the feasibility of a human-inspired sensorimotor framework based on discretized exploration and probabilistic command selection on an articulated robotic arm of RR type.
2. Analyze the influence of the time parameters used for actuator commands and sensory data.
3. Evaluate the effects induced by modifications of the robotic arm mechanics (friction in actuators and addition of series springs) after re-exploration on the modified system.

The paper is organized as follows: Section 2 describes the model on which the controller is implemented, explains the controller's working principle, and defines the performance metric used in this paper. Section 3 presents the results obtained for different types of trajectories and examines the influence of the time parameters, the actuator frictions, and the presence of series springs in the system in terms of tracking performance and framework feasibility and redeployability after re-exploration. Section 4 discusses the main limitations of this controller and proposes several solutions. Finally, Section 5 summarizes the findings and outlines directions for future research.

## 2 Methods

### 2.1 Model

**Figure 1:**
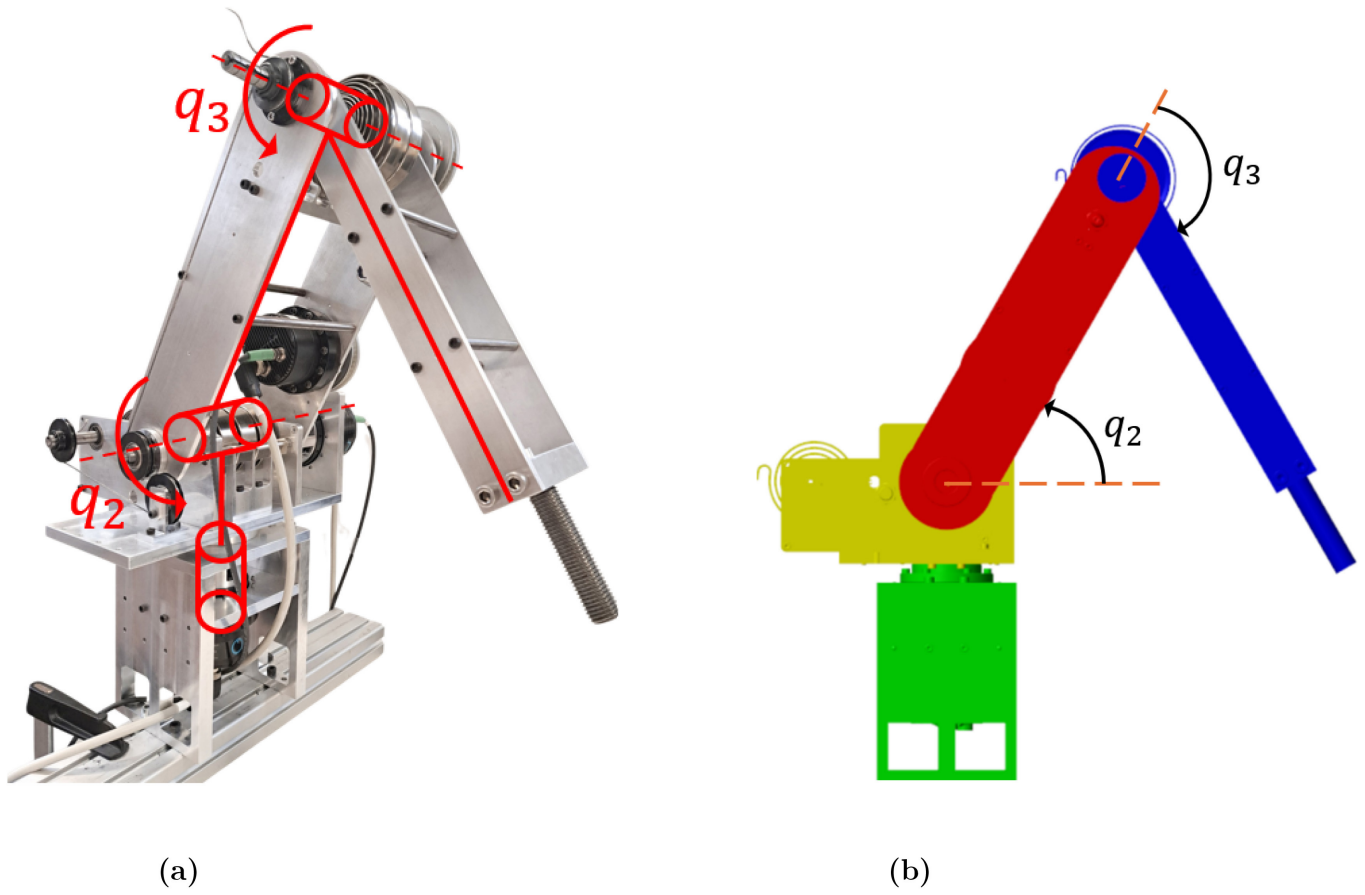
Articulated robotic arm of RRR type. **(a)** Reference prototype. **(b)** Simulated robotic arm.

The robotic arm considered in this paper is shown in Figure 1a and is the same as the one in Marchal et al. (2023). Nevertheless, only the joints of the robotic arm that are influenced by gravity are studied in this paper. Therefore, the first joint is not considered here since its axis of rotation is parallel to the direction of gravity. In other words, the robotic arm considered in this paper is a two-link manipulator of RR type. The mechanical parameters of this robotic arm are shown in Table 1. The robot is simulated in Simulink using Simscape Multibody (see Figure 1b). The solver used is ode1be (Backward Euler) because this solver is suited for abrupt changes or discrete events, being here the case due to the piecewise constant shape of the actuator currents as it will be explained in Section 2.2.2.

**Table 1:** Mechanical parameters of the robotic arm.

	Link 2	Link 3
*m* (*kg*)	3.940	1.960
*l* (*m*)	0.3	0.3
*l*_*c*_ (*m*)	0.0842	0.1205
*I*_*xx*_ (*kg m*^2^)	1.62*e*−2	5.09*e*−3
*I*_*xy*_ (*kg m*^2^)	1.8*e*−4	−4*e*−5
*I*_*xz*_ (*kg m*^2^)	5.36*e*−3	−1.13*e*−3
*I*_*yy*_ (*kg m*^2^)	4.38*e*−2	4.01*e*−2
*I*_*yz*_ (*kg m*^2^)	−2*e*−3	0
*I*_*zz*_ (*kg m*^2^)	3.09*e*−2	3.58*e*−2

*m* is the mass, *l* is the length, *l*_*c*_ is the distance between the joint and the center of gravity of the corresponding link, and *I*_*ij*_ (*i* = *x, y, z* and *j* = *x, y, z*) are the inertia parameters with respect to the center of gravity of the corresponding link.

### 2.2 Sensorimotor learning

The developed human-inspired controller consists of two phases, namely an exploration phase and an exploitation phase. The objective of the exploration phase is to build a discretized empirical state–action–response database linking sampled system states, actuator commands, and observed responses. To better reflect the characteristics of human motor dynamics adaptations, the following choices are made:

- The only sensory data learned is velocity in order to align with various studies showing that rhythmic adaptations remain possible with peripheral vision (Schmidt et al., 2007; Richardson et al., 2005) or using other sensory inputs which does not provide information on specific positions, such as auditory data (Shockley et al., 2007).
- The model does not know the system parameters before learning.
- Learning is carried out directly using the current sent to the actuators as motor command data.

In this work, the learned response representation is based on observed joint velocity responses. However, position information is not absent from the controller: joint positions are used together with joint velocities to define the sampled initial states during exploration, and they are also used during exploitation for current state matching.

#### 2.2.1 Exploration phase

The exploration phase involves collecting system response data to characterize the robotic arm's behavior over the sampled operating range and to populate the discretized empirical database used during exploitation. Specifically, this phase is structured into three sequential steps that are repeated for *n* iterations:

1. The robotic arm is placed in an initial state *s*_*i*_, defined by specific initial joint positions and speeds. For this purpose, the following sets are defined:

$$\begin{array}{l} Q_{2,0}=\left\{q_{2,0,1},q_{2,0,2},\ldots ,q_{2,0,n_{{\text{q}}_{2}}}\right\},\\ Q_{3,0}=\left\{q_{3,0,1},q_{3,0,2},\ldots ,q_{3,0,n_{{\text{q}}_{3}}}\right\},\\ {\dot{Q}}_{2,0}=\left\{{\dot{q}}_{2,0,1},{\dot{q}}_{2,0,2},\ldots ,{\dot{q}}_{2,0,n_{{\dot{\text{q}}}_{2}}}\right\},\\ {\dot{Q}}_{3,0}=\left\{{\dot{q}}_{3,0,1},{\dot{q}}_{3,0,2},\ldots ,{\dot{q}}_{3,0,n_{{\dot{\text{q}}}_{3}}}\right\} \end{array}$$ (1)

The set of all possible initial states is then given by:


$$\begin{array}{l} S=Q_{2,0}\times Q_{3,0}\times {\dot{Q}}_{2,0}\times {\dot{Q}}_{3,0} \end{array}$$ (2)

and each state is an element of this set, i.e. $s_{i}\in S$, with *i* = 1, 2, …, *n*_s_, where $n_{\text{s}}=n_{{\text{q}}_{2}}\cdot n_{{\text{q}}_{3}}\cdot n_{{\dot{q}}_{2}}\cdot n_{{\dot{q}}_{3}}$.

2. Both actuators are actuated with a certain constant amount of current, which can be different for both actuators. By defining:

$$\begin{array}{l} I_{2}=\left\{I_{2,1},I_{2,2},\ldots ,I_{2,n_{{\text{I}}_{2}}}\right\},\\ I_{3}=\left\{I_{3,1},I_{3,2},\ldots ,I_{3,n_{{\text{I}}_{3}}}\right\} \end{array}$$ (3)

the set of all possible current inputs is then:


$$\begin{array}{l} U=I_{2}\times I_{3} \end{array}$$ (4)

and each input vector corresponds to a pair of currents chosen from these sets, i.e. $u_{j}\in U$ with *j* = 1, 2, …, *n*_c_, where *n*_c_ = *n*_I_2__·*n*_I_3__.

3. The observed joint velocities (${\dot{q}}_{2,\text{obs},i,j}$, and ${\dot{q}}_{3,\text{obs},i,j}$) are stored during 0.1*s* with a sampling interval of 1*ms*.

These three steps are repeated for each possible combination of initial states *s*_*i*_ and actuator commands *u*_*j*_, namely *n*_s_·*n*_c_ = *n* iterations.

In other words, the exploration space is defined by the Cartesisan product of the discretized initial-state grid in Equation 1 and Equation 2 and the discretized input grid in Equation 3 and Equation 4.

It is also important to note that the exploration phase must be carefully performed by selecting appropriate initial states *s*_*i*_ and actuator current values *u*_*j*_. This selection process is nontrivial, as only a limited number of initial states and actuator current combinations can be tested to avoid dimensionality problems. As a result, the exploration strategy was determined through trial and error, ensuring a balance between a manageable number of explorations and sufficient tracking performance over a wide operating range of the robotic arm. The final exploration phase sampling configuration is presented in Table 2 with the value of *t*_obs_ initially set to 0.01*s*, but this will be discussed in Section 3.2.

**Table 2:** Sampled variables during the exploration phase.

$q_{2,\text{0}}({}^{\circ})$	15:15:75
$q_{3,\text{0}}({}^{\circ})$	–135:15:–15
${\dot{q}}_{2,\text{0}}({}^{\circ}/s)$	–60:10:60
${\dot{q}}_{2,\text{0}}({}^{\circ}/s)$	–60:10:60
*I*_2_ (A)	–3.5:0.2:3.5
*I*_3_ (A)	–2.5:0.2:2.5
Number of iterations	7 597 395

0A have also been explored for both joints.

In conclusion, the exploration phase provides insight into the system's dynamic response for a non-exhaustive set of actuator current values in both joints, and that for a non-exhaustive set of initial states. This process characterizes the system's behavior within the tested range and constructs a discretized empirical state–action–response database. No explicit analytical model of the dynamics is derived.

#### 2.2.2 Exploitation phase

The second phase, namely the exploitation phase, can begin once the exploration phase has been performed. The exploitation phase utilizes the knowledge gained from the exploration phase to control the robotic arm presented in Section 2.1. During exploitation, the controller does not update a continuous model online. Instead, it matches the current state to the closest explored state and selects the actuator command using a probabilistic maximum a posteriori rule over the discretely sampled responses.

Essentially, the objective of this exploitation phase is to dynamically adapt to desired joint speeds ${\dot{q}}_{d}$ based on external stimuli. This phase consists of six sequential steps, executed at regular intervals, referred to in this paper as the control time step (*t*_imp_):

1. The actual state is observed, namely the position and speed of both joints at the current time.
2. The closest state *s*_*i*_, that has been considered during the exploration phase, to the actual robotic arm's state is selected. Accordingly, although the exploration phase stores velocity-based response information, the online controller uses both joint positions and joint velocities to identify the nearest explored state.
3. For each joint and each combination of actuator commands *u*_*j*_ considered during the exploration phase, the normal distribution a posteriori associated with the state *s*_*i*_ is computed:

$$\begin{array}{l} \overset{\text{A posteriori}}{\overset{︷}{P\left(u_{2,j}|{\dot{q}}_{2,\text{d}}\right)}}=\frac{\overset{\text{Likelihood}}{\overset{︷}{P\left({\dot{q}}_{2,\text{d}}|u_{2,j}\right)}}\overset{\text{A priori}}{\overset{︷}{P\left(u_{2,j}\right)}}}{\underset{\text{Normalization}}{\underset{︸}{\overset{n_{\text{c}}}{\underset{k=1}{\sum }}P\left({\dot{q}}_{2,\text{d}}|u_{2,k}\right)P\left(u_{2,k}\right)}}},\\ \overset{\text{A posteriori}}{\overset{︷}{P\left(u_{3,j}|{\dot{q}}_{3,\text{d}}\right)}}=\frac{\overset{\text{Likelihood}}{\overset{︷}{P\left({\dot{q}}_{3,\text{d}}|u_{3,j}\right)}}\overset{\text{A priori}}{\overset{︷}{P\left(u_{3,j}\right)}}}{\underset{\text{Normalization}}{\underset{︸}{\overset{n_{\text{c}}}{\underset{k=1}{\sum }}P\left({\dot{q}}_{3,\text{d}}|u_{3,k}\right)P\left(u_{3,k}\right)}}} \end{array}$$ (5)

with *j* = 1, 2, …, *n*_c_, and where, as a reminder, *n*_c_ is the number of different actuator current values *u*_*j*_ considered during the exploration phase associated with the initial state *s*_*i*_.

4. The distribution a posteriori of each joint defined in Equation 5 is multiplied one to the other to obtain the distribution a posteriori of the system:

$$\begin{array}{l} P\left(u_{j}|{\dot{q}}_{d}\right)=P\left(u_{2,j}|{\dot{q}}_{2,\text{d}}\right)P\left(u_{3,j}|{\dot{q}}_{3,\text{d}}\right) \end{array}$$ (6)

with *j* = 1, 2, …, *n*_c_.

5. The maximum a posteriori of Equation 6 is computed, namely $\max\left(P\left(u_{j}|{\overset{{}^{\circ}}{q}}_{d}\right)\right)$, corresponding to the actuator commands most likely to achieve the desired joint speeds.
6. The commands *u*_*j*_ associated with the maximum computed in the previous step are sent to the actuators.

Note that the selected actuator commands are kept constant until the next control time step. This means that the actuator commands have always the shape of a piecewise-constant function.

It is also important to mention that since Equation 5 is related to probabilities, eight constraints (four for each joint) need to be imposed, as summarized in Equation 7:


$$\begin{array}{c} 0\leq P\left(u_{2,j}|{\dot{q}}_{2,\text{d}}\right)\leq 1\text{\&}0\leq P\left(u_{3,j}|{\dot{q}}_{3,\text{d}}\right)\leq 1,\\ \overset{n_{\text{c}}}{\underset{j=1}{\sum }}P\left(u_{2,j}|{\dot{q}}_{2,\text{d}}\right)=1\text{\&}\overset{n_{\text{c}}}{\underset{j=1}{\sum }}P\left(u_{3,j}|{\dot{q}}_{3,\text{d}}\right)=1,\\ 0\leq P\left(u_{2,j}\right)\leq 1\text{\&}0\leq P\left(u_{3,j}\right)\leq 1,\\ \overset{n_{\text{c}}}{\underset{j=1}{\sum }}P\left(u_{2,j}\right)=1\text{\&}\overset{n_{\text{c}}}{\underset{j=1}{\sum }}P\left(u_{3,j}\right)=1 \end{array}$$ (7)

with *j* = 1, 2, …, *n*_c_.

Furthermore, in the absence of prior knowledge, *P*(*u*_2, *j*_) and *P*(*u*_3, *j*_) are equiprobable, meaning that:


$$\begin{array}{l} P\left(u_{2,j}\right)=\frac{1}{n_{\text{c}}}\;\text{and }P\left(u_{3,j}\right)=\frac{1}{n_{\text{c}}} \end{array}$$ (8)

with *j* = 1, 2, …, *n*_c_.

Equation 8 implies that *P*(*u*_2, *j*_) and *P*(*u*_2, *k*_) (and respectively *P*(*u*_3, *j*_) and *P*(*u*_3, *k*_)) in Equation 5 can be omitted, meaning only the likelihood term is essential. As a result, Equation 5 can be rewritten as Equation 9:


$$\begin{array}{l} P\left(u_{2,j}|{\dot{q}}_{2,\text{d}}\right)=\frac{P\left({\dot{q}}_{2,\text{d}}|u_{2,j}\right)}{\sum_{k=1}^{n_{\text{c}}}P\left({\dot{q}}_{2,\text{d}}|u_{2,k}\right)},\\ P\left(u_{3,j}|{\dot{q}}_{3,\text{d}}\right)=\frac{P\left({\dot{q}}_{3,\text{d}}|u_{3,j}\right)}{\sum_{k=1}^{n_{\text{c}}}P\left({\dot{q}}_{3,\text{d}}|u_{3,k}\right)} \end{array}$$ (9)

with *j* = 1, 2, …, *n*_c_.

The likelihood term, which represents the probability of reaching the desired speed $\dot{q}d$ given the learned speed $\dot{q}\text{obs},i,j$ associated with the actuator commands *u*_*j*_, is assumed to follow a normal distribution centered on the desired speed for both joints. This assumption is justified by the observation that actuator behavior typically exhibits Gaussian-like variability due to sensor noise, friction, and other stochastic effects. Moreover, the normal distribution provides a smooth and tractable probabilistic relationship between desired and observed speeds, which facilitates analytical derivation and optimization. This modeling choice is consistent with standard practice in probabilistic robotics and control. Therefore, the likelihood is expressed as Equation 10:


$$\begin{array}{l} P\left({\dot{q}}_{2,\text{d}}|u_{2,j}\right)=\frac{1}{\sqrt{2\pi \sigma_{2}}}e^{\frac{-{\left({\dot{q}}_{2,\text{d}}-{\dot{q}}_{2,\text{obs},i,j}\right)}^{2}}{2\sigma_{2}^{2}}},\\ P\left({\dot{q}}_{3,\text{d}}|u_{3,j}\right)=\frac{1}{\sqrt{2\pi \sigma_{3}}}e^{\frac{-{\left({\dot{q}}_{3,\text{d}}-{\dot{q}}_{3,\text{obs},i,j}\right)}^{2}}{2\sigma_{3}^{2}}} \end{array}$$ (10)

with *j* = 1, 2, …, *n*_c_, and where σ_2_ and σ_3_ are standard deviations. They have both been set to 1 to ensure that different observed joint speeds can be distinguished while maintaining a smooth likelihood function. This value was empirically determined by trial and error. Note that the index *i* for ${\dot{q}}_{2,\text{obs},i,j}$ and ${\dot{q}}_{3,\text{obs},i,j}$ is the index that corresponds to the selected closest state *s*_*i*_ in the second step.

### 2.3 Performance evaluation

To evaluate the tracking performance of this controller, an appropriate performance index must be established. While the controller primarily tracks joint velocities, verifying speed tracking alone is insufficient, as accurate velocity tracking does not necessarily guarantee accurate position tracking. Furthermore, achieving balanced tracking performance across both joints is essential, rather than having near-perfect accuracy for one joint while the other performs poorly. Therefore, the performance index must account for both position and speed errors in both joints. In this study, we define the Geometric Global Error (GGE) as a performance metric, calculated as the geometric mean of the root-mean-square error (RMSE) for joint positions and speeds. Mathematically, it is expressed as Equation 11:


$$\begin{array}{l} \text{GGE}=(\prod_{i=2}^{3}\sqrt{\frac{\sum_{t=1}^{N}|q_{i,\text{d}}(t)-q_{i}(t){|}^{2}}{N}}\\ \;{\sqrt{\frac{\sum_{t=1}^{N}|{\dot{q}}_{i,\text{d}}(t)-{\dot{q}}_{i}(t){|}^{2}}{N}})}^{\frac{1}{4}} \end{array}$$ (11)

where *N* is the number of time steps. Note that the position and speed values should be expressed in radians.

While GGE provides a compact scalar summary of tracking quality, it compresses several error sources into a single value. Therefore, in the following results, per-joint position and speed errors are also examined, alongside GGE when needed, to determine whether a given performance difference mainly arises from position tracking, speed tracking, or imbalance across joints.

## 3 Results

### 3.1 Dynamics adaptation performance

As a reminder, the primary objective of this controller is to dynamically adapt to external stimuli. In the context of simulations, these external stimuli correspond to predefined desired joint speeds. To demonstrate the effectiveness of the proposed controller, two different desired trajectories are considered: Sinusoidal and trapezoidal velocity profile trajectories.

#### 3.1.1 Sinusoidal trajectory

**Figure 2:**
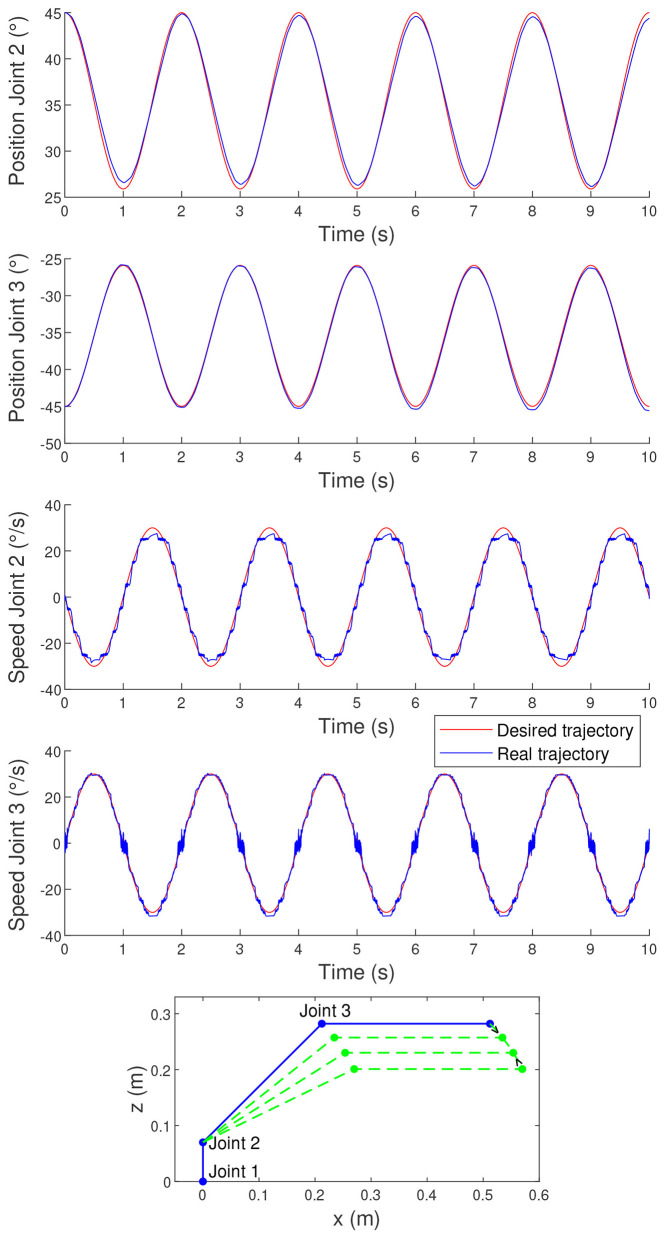
Tracking performance for sinusoidal motion. GGE = 0.0152, with RMSE (*q*_2_) = 0.0117*rad*, RMSE (*q*_3_) = 0.0093*rad*, RMSE (${\dot{q}}_{2}$) = 0.0302*rad*/*s*, and RMSE (${\dot{q}}_{3}$) = 0.0162*rad*/*s*. The last plot shows the robotic arm motion.

The desired joint speeds for a sinusoidal motion are expressed by:


$$\begin{array}{l} {\dot{q}}_{2,\text{d}}=-A\sin\left(2\pi ft\right)\\ {\dot{q}}_{3,\text{d}}=A\sin\left(2\pi ft\right) \end{array}$$ (12)

where for the first validation test, *A* is set to 30°/*s* and *f* to 0.5*Hz*. The initial position of the joints has been set, respectively, to 45° and −45°, and the time vector *t* goes from 0 to 10*s* with a step of 1*ms*. The amplitude and frequency of the joint motions were selected to avoid limit conditions and to remain within the range of initial states considered during the exploration phase. The tracking performance for this desired trajectory is illustrated in Figure 2. The GGE for this motion is 0.0152, indicating a decent level of accuracy. The corresponding per-joint RMSE values are reported in Figure 2 to complement this aggregate metric. These values show that the largest contribution comes from the velocity tracking error of Joint 2, while the position errors remain smaller. Overall, these error levels remain acceptable in scenarios where the objective is to adapt dynamically to external stimuli, rather than achieving precise spatio-temporal trajectory tracking.

**Figure 3:**
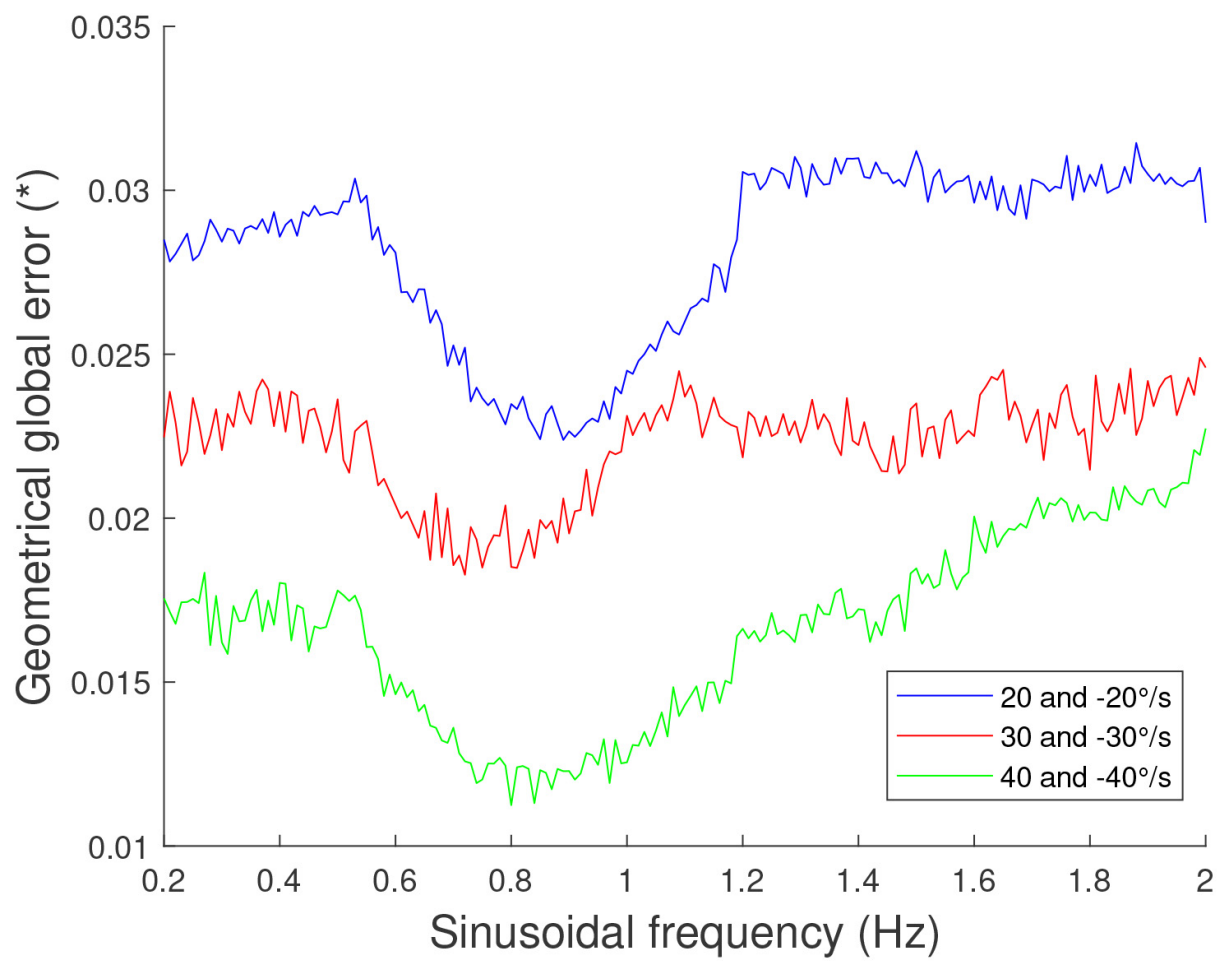
Frequency and amplitude analysis for sinusoidal motions. The two values in the legend are the amplitudes for Joints 2 and 3, respectively.

However, the trajectory tracking shown in Figure 2 is only for one specific sinusoid. To allow further analysis, Figure 3 presents the results for sinusoidal motions at varying frequencies (*f*) for 3 distinct combinations of amplitudes (*A*), and with initial joint positions set at 15° and −15° for Joints 2 and 3, respectively.

The findings indicate that the GGE is higher for small amplitudes, which aligns with expectations. Indeed, for low amplitudes, the joint speed variations are minimal. This means that the duration of the speed steps, which are clearly visible in the speed profile of Joint 2 in Figure 2, is increasing. These prolonged speed steps result in reduced tracking accuracy, thereby increasing the GGE. It is important to note that these steps primarily stem from the limited number of initial speeds tested during the exploration phase, which induces an under-sampling of the possible actuator commands.

Furthermore, for each combination of amplitudes, the controller exhibits preferential tracking frequencies, which remain relatively consistent across the three distinct combinations of amplitudes considered. This trend was anticipated, as preferential frequencies are mostly independent of amplitude in the absence of extreme motion conditions. Minor variations in these preferential frequencies can be attributed to the system's inherent nonlinearity, where motion amplitude subtly influences dynamic properties.

Additionally, the results reveal a slight increase of the GGE at both relatively low and relatively high frequencies, regardless of amplitude. This behavior can be explained by an analysis of the controller's working principle and the nature of sinusoidal motions. At low frequencies, the relatively small increase of the GGE arises due to the increased duration of speed steps, as for the low amplitudes. At high frequencies, the relatively small increase of the GGE can be attributed to the greater number of sinusoidal cycles within a given time frame. As a result, the acceleration changes sign more frequently (corresponding to the speed minima and maxima), where tracking errors are most pronounced, as seen in the speed profile of Joint 2 in Figure 2. Moreover, as frequency increases, the number of instances where the joint speeds reach zero also rises. Figure 2 shows that for Joint 3, speed tracking errors are particularly noticeable at these zero-speed transitions.

In conclusion, the controller demonstrates improved performance for specific sinusoidal frequencies, aligning with the system's natural dynamics. In addition, the tracking is also better for desired sinusoidal velocities with high amplitudes. Nevertheless, it still achieves decent tracking performance across a wide range of sinusoidal frequencies and amplitudes.

#### 3.1.2 Trapezoidal velocity profile trajectory

Another interesting type of motion to consider is the trapezoidal velocity profile trajectory commonly used in robotics. A key characteristic of this trajectory is the presence of a constant velocity phase, distinguishing it from a sinusoidal trajectory. Evaluating the controller's performance on this trajectory provides insight into its ability to maintain a constant joint speed over a given duration. Mathematically, the desired speed for this trajectory is defined as follows:


$${\dot{q}}_{\text{d}}=\{\begin{array}{ll} \frac{v_{\text{m}}}{t_{\text{a}}}t, & t\in [0,t_{\text{a}}]\\ v_{\text{m}}, & t\in \;]t_{\text{a}},t_{\text{f}}\text{​}-\text{​}t_{\text{d}}]\\ v_{\text{m}}-\frac{v_{\text{m}}}{t_{\text{a}}}(t\text{​}+\text{​}t_{\text{d}}\text{​}-\text{​}t_{\text{f}}), & t\in \;]t_{\text{f}}\text{​}-\text{​}t_{\text{d}},t_{\text{f}}] \end{array}$$ (13)

where *v*_m_ corresponds to the speed in the constant velocity phase, *t*_a_ = 0.1*s* is the duration of the acceleration phase, *t*_d_ = 0.1*s* is the duration of the deceleration phase, and *t*_f_ = 1*s* is the total duration of the motion. To analyze the controller's ability to track different constant joint speeds, this trajectory is repeated over 10 iterations with 10 different values for *v*_m_, for each joint.

**Figure 4:**
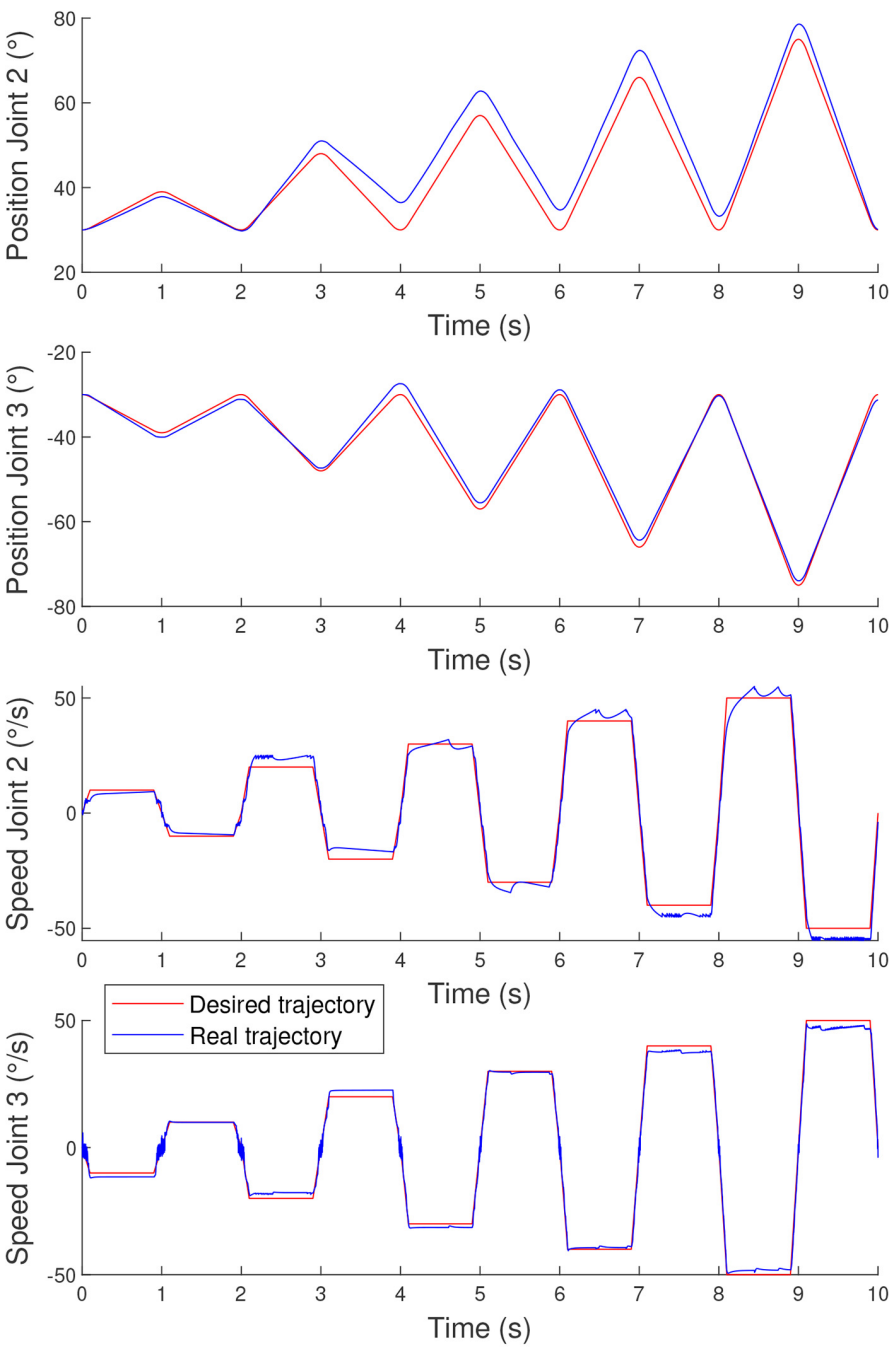
Tracking performance for trapezoidal velocity profile motion. GGE = 0.0417, with RMSE (*q*_2_) = 0.0953*rad*, RMSE (*q*_3_) = 0.0196*rad*, RMSE (${\dot{q}}_{2}$) = 0.0414*rad*/*s*, and RMSE (${\dot{q}}_{3}$) = 0.0391*rad*/*s*.

The results presented in Figure 4 indicate that the controller has a little more difficulty maintaining constant joint speeds than following variable joint speeds. The corresponding per-joint RMSE values are reported in Figure 4 to complement the aggregate GGE. These values show that the largest contribution comes from the position tracking error of Joint 2. This is consistent with the fact that, for trapezoidal velocity profiles, achieving and maintaining the exact desired constant joint speeds proves more challenging, which in turn leads to larger accumulated position errors over time. This observation was expected since the nature of the controller is not to be as stable as possible, but to be as reactive as possible.

### 3.2 Influence of the time parameters

**Figure 5:**
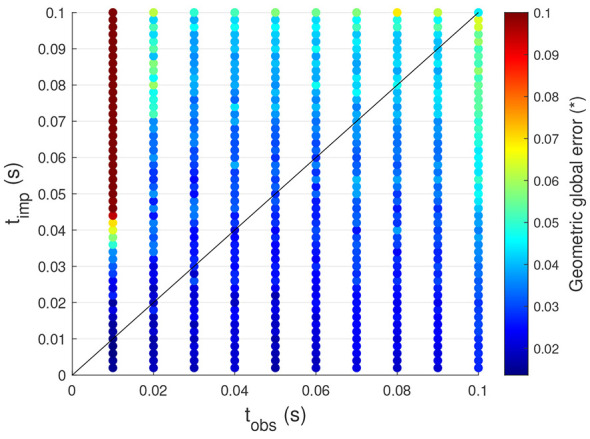
Influence of the time parameters on the tracking performance of the controller for sinusoidal motions.

Two key time parameters play a crucial role in the proposed controller. The first is the observation time (*t*_obs_), which defines the time instant at which the observed joint speeds from the exploration phase are selected for use in the exploitation phase. The second is the control time step (*t*_imp_), which determines the fixed interval at which the steps of the exploitation phase are executed. To better understand their impact on tracking performance, an analysis is conducted by tracking the same trajectory as in Section 3.1.1 (sinusoidal trajectory defined by Equation 12), while systematically varying *t*_obs_ and *t*_imp_. The results of this investigation are presented in Figure 5.

One can observe that as the control time step increases, the GGE also increases. This behavior is expected since a larger control time step results in less frequent updates to the system, leading to greater tracking errors. Therefore, when high tracking accuracy is required, the control time step should be kept small. Moreover, when the control time step *t*_imp_ exceeds the observation time *t*_obs_, the GGE becomes even larger. This effect is particularly pronounced when the difference between the two parameters is significant, as shown in Figure 5 for small observation times and large control time steps. This outcome is expected because the system likely achieves the desired joint speed, but due to the delayed update (high *t*_imp_), the arm continues to move and deviates from the desired trajectory. Consequently, the control time step should always be smaller than the observation time to prevent excessive deviations.

Additionally, even when the control time step remains smaller than the observation time, increasing the observation time also leads to a higher GGE. This is because a larger observation time increases the discrepancy between the learned joint speeds during the explorations and the selected observed speeds, reducing the likelihood of finding observed speeds that closely match the desired speeds. Thus, to maintain good tracking performance, the observation time should not be too large.

In conclusion, to achieve the best tracking performance, the observation time and control time step should be not too high, with the control time step always smaller than the observation time. However, excessively reducing the control time step has a drawback. Indeed, it limits the exploitation of the system's natural dynamics and, as the system is controlled more frequently, potentially increases the computational demand.

**Figure 6:**
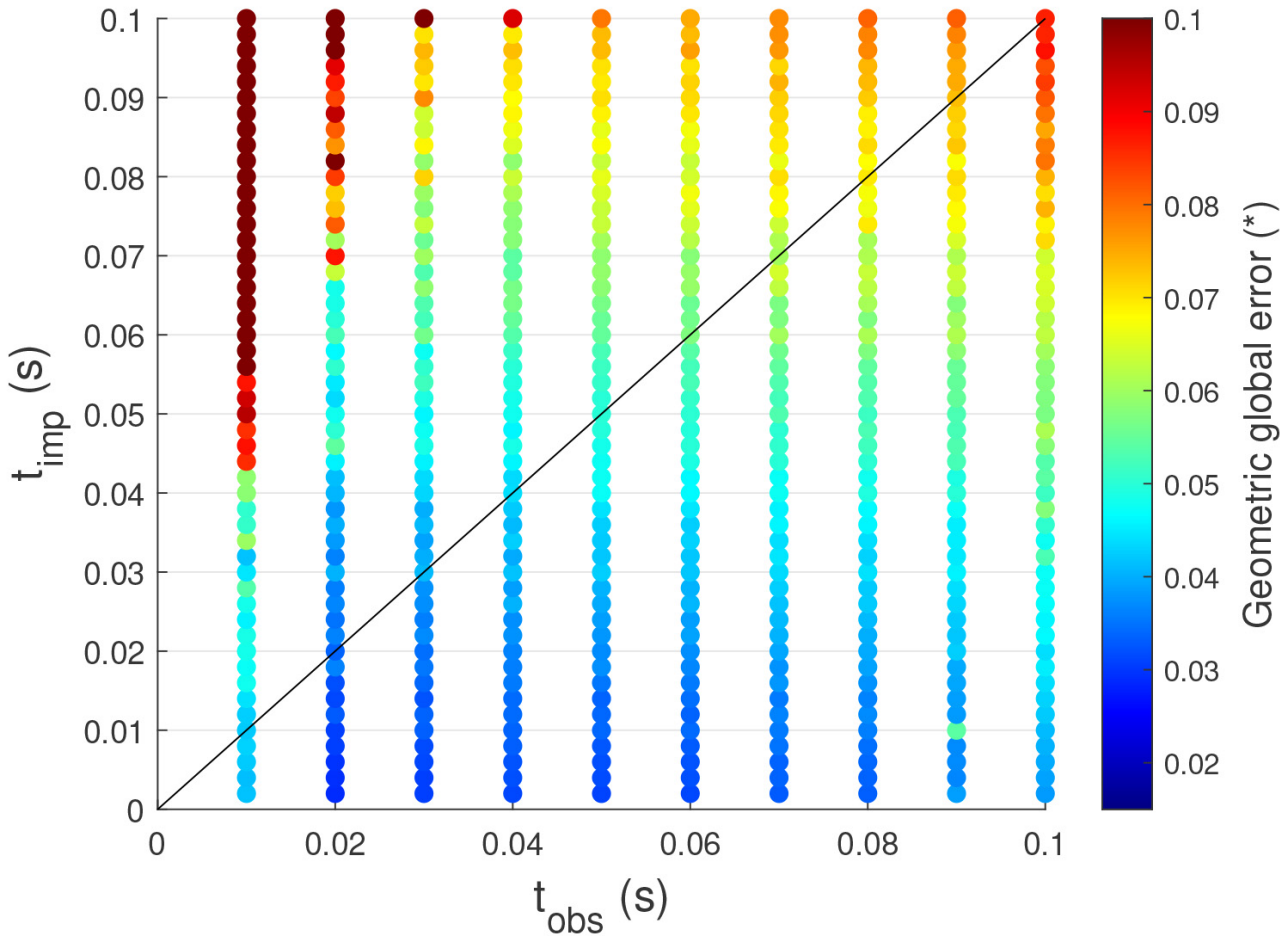
Influence of the time parameters on the tracking performance of the controller for trapezoidal velocity profile motions.

However, this conclusion applies specifically to motions in which the joint speeds are not kept constant, such as sinusoidal trajectories. Conversely, when the same analysis is applied to motions where the joint speeds remain constant at certain moments, the results yield partially different conclusions. Figure 6 presents the findings of this analysis, this time using the trapezoidal velocity profile trajectory defined in Equation 13 and illustrated in Figure 4 as the desired trajectory.

The assumption that smaller observation times always lead to better tracking performance no longer holds in this case. Specifically, the best tracking performance is achieved at *t*_obs_ = 0.02*s* rather than 0.01*s*. This can be attributed to the fact that when *t*_obs_ is very small, the control currents applied to the actuators are more likely to deviate from the previous values to achieve the desired joint speeds. These abrupt current variations induce sharp accelerations, resulting in a less stable system in which maintaining constant joint speeds becomes more challenging. However, aside from this difference, the previous conclusions remain valid.

### 3.3 Influence of the mechanical system parameters

#### 3.3.1 Influence of the friction in the actuators

As described in Marchal et al. (2024), harmonic drive actuators have been used to control the robotic arm. However, these actuators exhibit significant Coulomb and viscous friction, a common characteristic of actuators with high transmission ratios and harmonic drives. Such properties are common for actuators designed for robotic systems requiring high transmission ratios and harmonic drives (Hamon et al., 2010).

Furthermore, actuators with high friction significantly hinder the exploitation of the natural dynamics of the system (Verstraten et al., 2016). Effective utilization of natural dynamics requires the system to move substantially under gravity or inertia alone, without actuator input. This is known as a backdrivable system. Excessive Coulomb and viscous friction in the actuators can impede such motion, making the system non-backdrivable. In some robotic arms, even when positioned horizontally, namely where the gravitational torque is maximal, their movement may remain restricted due to the high frictional forces within the actuators.

Therefore, replacing harmonic drive actuators with planetary gearbox actuators, which have lower friction losses, would improve dynamic behavior (Hamon et al., 2010) and may change results. The modeling of such actuators is detailed in Furnémont et al. (2018). In this paper, actuators with high friction are defined as “HF Actuators,” and actuators with fewer friction losses are called “LF Actuators.”

**Figure 7:**
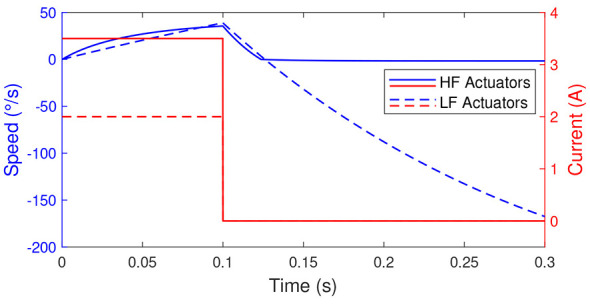
Natural dynamics exploitation of Joint 2 for the two types of actuators. HF stands for high-friction and LF for low-friction.

First, to illustrate why lower-friction actuators, and therefore backdrivable actuators, are necessary for exploiting natural dynamics, Figure 7 presents the system's dynamic response for both actuator types. For the results shown Figure 7, Joint 3 is fixed at 0°, while Joint 2 starts at 0°, meaning that the robotic arm starts in a horizontal position. Furthermore, a given current is applied to the actuator of Joint 2 for the first 0.1*s*. After this initial period, no current is applied, allowing observation of the system's natural dynamics.

The results show that when using an actuator with high friction (HF Actuators), the joint speed rapidly converges to nearly 0°/*s*. This indicates that, even under a high gravitational load, the system is unable to move freely without external power, preventing any significant exploitation of natural dynamics. Conversely, when using an actuator with lower friction losses (LF Actuators), the joint speed continues to decrease after the current is removed, driven by gravitational torque (and, to a lesser extent, inertia). Importantly, the speed does not converge to zero, demonstrating that in this case, the natural dynamics of the system can be effectively utilized.

Since the actuators (and consequently the system) have been modified, a new exploration phase is performed using the same robotic system with the modified actuators (LF actuators). Since the modified actuator dynamics require a new exploration phase in the present study, these results should be interpreted as demonstrating successful application of the framework after re-exploration on the modified plant. The variables sampling during this new exploration phase is outlined in Table 3 with adapted current values to account for the different actuator properties.

**Table 3:** Sampled variables during the exploration phase with LF actuators.

*q*_2, 0_(°)	15:15:75
*q*_3, 0_(°)	–135:15:–15
${\dot{q}}_{2,0}\left(./s\right)$	–60:10:60
${\dot{q}}_{3,0}\left(./s\right)$	–60:10:60
*I*_2_(*A*)	-0.5:0.05:2
*I*_3_(*A*)	-0.1:0.025:0.6
Explorations	11 247 795

**Figure 8:**
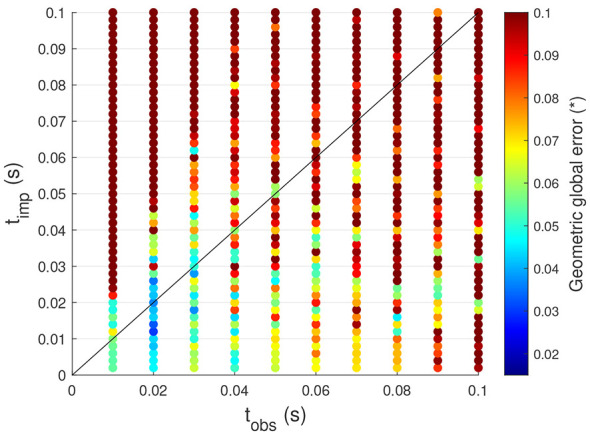
Influence of the time parameters on the tracking performance of the controller on the system with LF Actuators for sinusoidal motions.

Similar time parameter analysis conducted in Section 3.2 can be repeated to evaluate the tracking performance under these new conditions. The results are presented in Figure 8, where the same color scale as in Figure 5 has been maintained to facilitate direct comparison.

First, it can be observed that the tracking performance is generally worse than in the case of high-friction actuators, at almost all values of *t*_obs_ and *t*_imp_. However, this result is expected. With lower friction, the system becomes more sensitive to abrupt changes in actuator currents, which occur naturally when using this controller. In contrast, higher friction acts as a damping mechanism, reducing sudden accelerations and stabilizing the system's response to actuator inputs. Furthermore, with low-friction actuators, the effects of inertia become more pronounced. This is because friction dampens inertia's effects, meaning that when friction is low, the influence of acceleration becomes significant.

Another key difference is that smaller values of *t*_obs_ and *t*_imp_ no longer yield the best tracking performance, unlike in Section 3.2 for actuators with high friction. The optimal values are found to be *t*_obs_ = 0.02*s* and *t*_imp_ = 0.012*s*. The reason for this difference lies in how inertia effects are handled. Since initial accelerations are not considered in the exploration phase, only a partial representation of inertia's influence is captured. Therefore, when *t*_obs_ is too small, these missing inertia effects have a greater impact on the system's response, leading to poorer tracking performance. However, as *t*_obs_ increases, the effects of initial accelerations become less dominant, improving tracking accuracy. Nevertheless, setting *t*_obs_ too high is still not advisable, as previously discussed in Section 3.2.

#### 3.3.2 Implementation of springs

Adding springs to the robotic arm structure can provide several advantages, depending on their configuration. A common approach is to add springs between the actuators and the joints. This configuration is called Series Elastic Actuation (SEA). Among its various advantages, SEA improves the energy efficiency of robotic systems by reducing the speed requirements in case of interaction with the environment or high acceleration/deceleration phases (Verstraten et al., 2017). Implementing SEAs introduces additional compliance into the robotic arm structure, significantly altering the system dynamics. As a result, it is essential to investigate whether the time parameters influence the tracking performance in the same way as discussed in Section 3.2.

To explore this, series springs with stiffness values of 6*Nm*/*rad* and 1.25*Nm*/*rad* were implemented at Joints 2 and 3, respectively. These values were selected based on the maximum gravitational torque acting on both joints, which is approximately 12*Nm* for Joint 2 and 2.5*Nm* for Joint 3. The chosen stiffness ensures that spring deflection never exceeds 2 *rad*, preventing excessive deformation while maintaining sufficient compliance. Additionally, this selection avoids excessively high stiffness, which would result in behavior similar to rigid actuators, as in previous cases.

**Figure 9:**
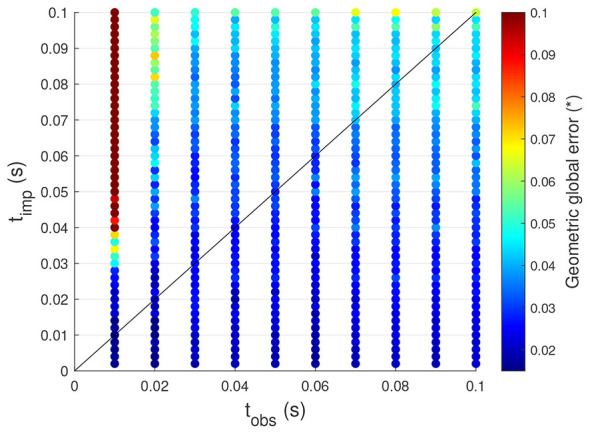
Influence of the time parameters on the tracking performance of the controller on the system with SEAs for sinusoidal motions.

To evaluate the system's behavior with SEAs, the same trajectory as in Section 3.1.1 is tracked. The results are presented in Figure 9. For a fair comparison, an exploration phase with sampled variables similar to the ones shown in Table 2 was conducted, but on a system that naturally incorporates SEAs in both joints. As in the low-friction actuator case, these results reflect successful application of the framework after an exploration phase performed on the modified system. As seen in Figure 9, the conclusions remain consistent with those drawn in Section 3.2, meaning that the controller can effectively handle systems with SEAs without issue.

Additionally, the electrical energy consumption of both systems, namely the one with rigid actuators and the one with SEAs, was compared, as SEAs are frequently implemented for energy efficiency purposes. However, no significant differences in energy consumption were observed. This result is consistent with expectations and can be explained by three main factors.

First, for smooth desired trajectories without disturbances, which is the case in this study, SEAs do not significantly reduce electrical energy consumption (Bolivar et al., 2019). This is because, under such conditions, the actuators do not experience large load torque variations that the series springs could smooth out to improve efficiency.

Second, SEAs are less effective when inertial torques are relatively small compared to gravitational torques, as seen in this system (Beckerle et al., 2017). When gravitational torques dominate and the motion does not involve significant acceleration and deceleration phases, the opportunity for energy storage and return within the series springs is minimal. As a result, SEAs provide little benefit in reducing energy consumption in this scenario.

Finally, for SEAs to effectively reduce electrical energy consumption, the desired trajectory should be reasonably close to the natural frequency of the series springs (Verstraten et al., 2016). However, in this study, the springs were not designed for a specific trajectory since the goal of this controller is to dynamically adapt to external stimuli, rather than being optimized for a single motion pattern. Designing series springs to match a particular trajectory would contradict this objective. For these three reasons, SEAs do not provide a significant reduction in electrical energy consumption in the studied system.

## 4 Discussion

The present paper proposes a sensorimotor learning-based controller in which the learned response representation during the exploration phase is deliberately based on observations of joint velocities. This choice aligns with findings from human studies, which suggest that rhythmic adaptations can be unintentional (spontaneous) and can occur even in the absence of explicit positional information (Issartel et al., 2007). Furthermore, as demonstrated in Mostafaoui et al. (2022), controlling a robot to emulate human-like rhythmic behavioral adaptation enhances the naturalness of human-robot interaction.

However, unlike in Mostafaoui et al. (2022), which relies on angular position control, learning is performed here based on current commands, which, in the case of a robotic system, can most closely resemble to muscle commands. It should be noted that few computational motor control models use direct learning on current commands. Although the exploration phase is intentionally based on velocity observations for biological inspiration and simplicity, joint positions and velocities are still used during exploitation to perform current state matching. However, it has limitations that we will discuss further.

First, as described by Schmidt et al. (2007), incorporating positional feedback (position tracking) could enhance motion adaptation to external visual stimuli. In such a case, a hybrid control scheme combining the current learning-based controller with, for example, a proportional feedback term, would be biologically inspired and practically effective (Therrien and Wong, 2022). Indeed, human motor control is known to integrate predictive and reactive components, and similar feedback corrections, akin to proportional control, could be applied without compromising the learning-based nature of the proposed framework (Mathew and Crevecoeur, 2021).

The choice to observe joint speeds at a specific observation time *t*_obs_ is motivated by simplicity, but alternative strategies, such as averaging over a given duration could also have been an option. In the present study, mechanical changes are handled by performing a new exploration phase on the modified system. Accordingly, the current results demonstrate successful redeployment of the framework after re-exploration rather than online adaptation without re-learning. Nevertheless, extending the framework toward online adaptation should be conceptually straightforward, since the controller relies on an empirical state–action–response database that could, in principle, be updated during exploitation as new states and responses are observed. Such an extension would not require a change in the overall control philosophy, but rather an enrichment of the learned database during operation.

Furthermore, the normal distribution a posteriori used during the exploitation phase assumes uniform prior probabilities in all the presented results. However, in the presence of prior knowledge, it is possible to incorporate non-uniform prior probabilities, such as those based on energy consumption, which can improve efficiency, especially in redundant or overactuated systems. Additionally, the a priori term in Equation 5 can also play a crucial role when the learning process is updated beyond the exploration phase, particularly during the exploitation phase.

The decision to express the desired dynamics as a combination of two desired velocities, namely one for each joint, is justified by simplicity. Indeed, another possibility could be to express the desired dynamics as the speed of the end-effector. Several questions can therefore be asked: Is the control responsible for interpersonal coordination (end-effector velocity adaptation) independent of the control responsible for intrapersonal coordination (coordination of the different arm joints and segments)? Should each joint be entrained rhythmically independently? The question of whether or not the control of interpersonal coordination overlaps with intrapersonal coordination is relevant since humans are capable of rhythmically coordinating themselves while performing different gestures. We could therefore imagine a system responsible for interpersonal coordination that would be juxtaposed with a system managing intrapersonal coordination. This question remains, for human motor control, widely debated (Degallier and Ijspeert, 2010; Schaal et al., 2004).

Analysis of tracking performance across sinusoidal frequencies revealed better performance at specific frequencies. This behavior parallels findings in human motor control, where individuals naturally prefer certain rhythmic frequencies that align with their natural dynamics, where spontaneous interpersonal coordination is favored (Richardson et al., 2005). Therefore, since this controller is designed to be human-inspired and to learn its inner dynamics from exploration, its performance variation across frequencies reflects an inherent preference for certain natural dynamics. This can explain the improved performance of this controller for specific sinusoidal frequencies.

A notable limitation of the current approach lies in the design of the exploration phase, which accounts solely for initial joint positions and velocities, while neglecting initial joint accelerations. In practice, this means that each exploration begins with zero joint accelerations, effectively assuming the system's initial inertia to be negligible. This simplification significantly reduces the dimensionality of the exploration space. However, it may adversely affect tracking performance, particularly in systems equipped with low-friction actuators where inertial effects play a more prominent role. During exploitation, the controller selects actuator commands based on data generated under the assumption of zero initial inertia, which is unlikely to hold in most real-world scenarios. As a result, the actuator commands may not yield joint velocities that align closely with the desired values, leading to degraded tracking accuracy. Incorporating initial joint accelerations into the exploration phase could mitigate this issue and improve control precision, though it would inevitably increase the computational burden and complexity of the learning process.

The observed sensitivity of tracking performance to the choice of time parameters underscores the importance of tuning these parameters in accordance with the system's physical characteristics. High-friction systems, which exhibit damped behavior, benefit from shorter control intervals, whereas low-friction or backdrivable systems require longer intervals to avoid instability induced by abrupt command changes. This principle also applies to humans, as they naturally adapt their motor control to their own physiological and neurological state, which depends on factors such as muscle strength, joint flexibility, and neural processing speed (Mulla and Keir, 2023; Enoka, 2015).

The discrete nature of the exploration phase imposes another important limitation: the current observed state during exploitation rarely matches the learned states exactly. This can lead to suboptimal actuator commands. Increasing the number of initial states and actuator current values considered during the exploration phase is not always practical due to time and memory constraints. Interpolation strategies, either across actuator currents or initial states, offer a promising compromise by enabling smoother command selection from coarsely sampled data. A further limitation concerns scalability. The present study is restricted to a 2-DOF RR arm, for which discretized exploration remains manageable. For higher-DOF or redundant manipulators, the size of the explored state and input spaces would grow rapidly, increasing the number of required exploration samples, storage requirements, and the sparsity of nearest-state matching during exploitation. Direct extension of the present implementation to higher-dimensional systems would therefore require additional mechanisms such as interpolation, local approximation, structured exploration, dimensionality reduction, or online updating of the learned database. More broadly, the present study is intended as a feasibility demonstration of the proposed framework on a low-dimensional robotic system under different motion and mechanical conditions, rather than as an extensive comparative benchmark against established control approaches.

## 5 Conclusion

In this study, we presented a human-inspired sensorimotor control framework based on offline exploration of sampled states and actuator commands, followed by online nearest-state matching and probabilistic command selection. On a 2-DOF RR robotic arm, the results demonstrate the feasibility of this approach across several motion profiles and mechanical configurations. The framework does not rely on an explicit analytical dynamics model, but it does use current joint positions and velocities during exploitation to identify the closest explored state. When the plant mechanics are modified, the present study applies the framework after a new exploration phase on the modified system. Accordingly, the current results demonstrate redeployability after re-exploration rather than experimentally validated online adaptation to changing mechanics. However, because the proposed controller is based on a learned empirical state-action-response representation, extending it toward online adaptation through database updates during exploitation should be feasible in principle without major changes to the framework. Key findings indicate that although the controller performs better for certain frequencies, it retains adaptability across a broad range of motions. Additionally, we show that the optimal values for the observation time and control time step are highly dependent on actuator friction. Systems with high friction benefit from minimal observation and control time steps, whereas those with lower friction require a balance between responsiveness and stability. However, due to its discrete nature, perfect tracking remains a challenge. To mitigate this, future developments may incorporate feedback integration, interpolation, and richer state representations.

In future work, we aim to enhance the controller by continuously updating the learned database during exploitation, allowing it not only to refine its knowledge as new states are encountered, but also to progressively extend the framework from redeployment after re-exploration toward online adaptation to changes in system dynamics caused by factors such as wear or environmental variations. We also plan to extend the framework to higher-dimensional and redundant robotic arms while addressing the associated growth of the explored state and input spaces, and to investigate energy-aware control strategies within the exploitation phase. Overall, this sensorimotor learning framework provides a promising, intuitive foundation for adaptive robotic control in real-world scenarios where model precision is difficult to guarantee.

## Data Availability

The raw data supporting the conclusions of this article will be made available by the authors, without undue reservation.
